# MTLD, a Database of Multiple Target Ligands, the Updated Version

**DOI:** 10.3390/molecules22091375

**Published:** 2017-09-06

**Authors:** Chao Chen, Meng Wu, Shan Cen, Jianhui Wu, Jinming Zhou

**Affiliations:** 1 North China Institute of Science and Technology, Beijing 065201, China; angelstudio@gmail.com; 2 Institute of Medicinal Biotechnology, Chinese Academy of Medical Science, Beijing 100050, China; wumeng_1915@163.com (M.W.); shancen@hotmail.com (S.C.); 3 Lady Davis Institute for Medical Research, Jewish General Hospital, Montreal, QC H3T 1E2, Canada; jian.h.wu@mcgill.ca

**Keywords:** polypharmacology, Multiple Target Ligands (MTLs), drug discovery, database

## Abstract

Polypharmacology plays an important role in drug discovery and polypharmacology drug strategies provide a novel path in drug design. However, to develop a polypharmacology drug with the desired profile remains a challenge. Previously, we developed a free web-accessible database called Multiple Target Ligand Database (MTLD, www.mtdcadd.com). Herein, the MTLD database has been updated, containing 2444 Multiple Target Ligands (MTLs) that bind with 21,424 binding sites from 18,231 crystal structures. Of the MTLs, 304 entries are approved drugs, and 1911 entries are drug-like compounds. Also, we added new functions such as multiple conditional search and linkage visualization. Through querying the updated database, extremely useful information for the development of polypharmacology drugs may be provided.

## 1. Introduction

Polypharmacology, which refers to a single drug acting on multiple targets through either a unique pathway or multiple pathways, is regarded as the main cause of severe side effects or toxicity of drugs [1,2,3]. Recently, owing to the exponential growth of molecular data and the rapid advancement in technologies, evidence is accumulating that polypharmacology is not only widespread, but also important for the efficacy of drugs [4,5,6]. For example, clozapine is the ‘gold standard’ atypical antipsychotic drug. It is thought to normalize glutamatergic and dopaminergic neurotransmission via complex interactions with a large number of molecular targets, which are probably responsible for its exceptionally beneficial actions in schizophrenia and related disorders [4]. Additionally, several highly efficient drugs, such as salicylate [7], metformin [8] or gleevec [9] enhance therapeutic efficacy by acting on multiple targets simultaneously. In particular, it is now generally accepted that the activity at a single receptor is insufficient for a complex disease involving multiple pathogenifc factors, such as cancer, diabetes, neurodegenerative syndrome, and cardiovascular diseases [10]. Thus, polypharmacology is recognized as a valuable new opportunity for drug discovery and development, opening novel avenues to rationally design the next generation of more effective, less toxic, therapeutic agents [11].

However, polypharmacological drug discovery remains a huge challenge, and current clinically applied polypharmacological drugs are mainly discovered at random. Rational drug design, which combines computational tools and structural information, has become the most promising and attractive strategy in polypharmacological drug discovery [12,13]. The structures from Protein Data Bank (PDB) supply detailed 3D information of the ligand and protein, which could provide quite useful information for the rational design of polypharmacological drugs. Recent research in polypharmacology, based on the 3D structures in PDB, indicates that the polypharmacology of a drug may be mainly due to the similarity of protein binding sites, as well as the molecular complexity of a ligand [14,15]. Previously, we constructed a database termed Multiple Target Ligand Database (MTLD, www.mtdcadd.com), extracted from the PDB database (Version: December 2012, San Diego, CA, USA) [16]. Since the online launch of the MTLD database, the MTLD was visited and queried more than 1000 times. In this work, the MTLD was updated, and two novel functions were added, and we hope that the new version of MTLD may provide more useful information for the development of polypharmacological drugs.

## 2. Results

### 2.1. The Mining of the MTLs from PDB

**Figure 1:**
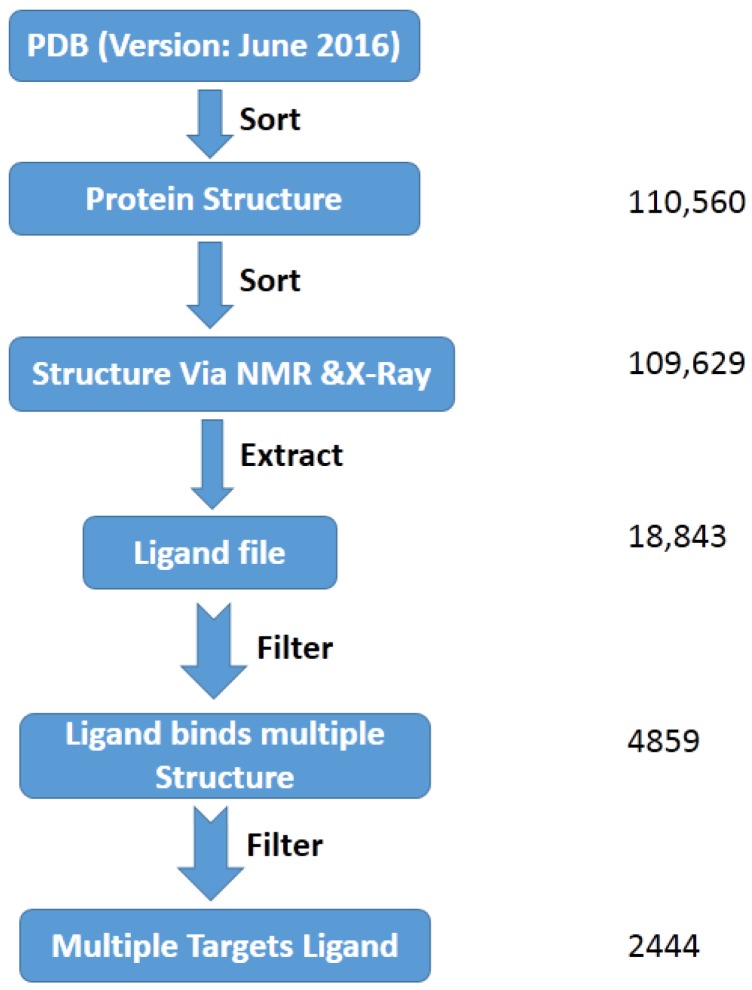
Dataset collection of the MTLD.

The PDB archive (version: June 2016) was applied for data mining in the update of the MTLD. As a result (Figure 1), all 110,560 protein structures were collected from PDB. Among these structures, 109,629 structures were solved through NMR and X-Ray methods. 91,864 ligand coordinate files were obtained, which contained 18,843 unduplicated ligands. 85,805 binding site coordinate files were outputted. Among these ligands, 4859 ligands were found to bind to more than one PDB structure. After removing the redundancy of crystal structural entries bound to the same ligand (the sequence identity between protein pairs was restricted to <35%), 2444 MTLs (≈13.0% of total unduplicated extracted ligands) were extracted from the PDB and archived in the MTLD, which bind with 21,424 binding sites from 18,231 crystal structures. The updated MTLD contains 41% more MTLs than the previous version of MTLD (Table 1).

### 2.2. Statistics for MTLD

**Figure 2:**
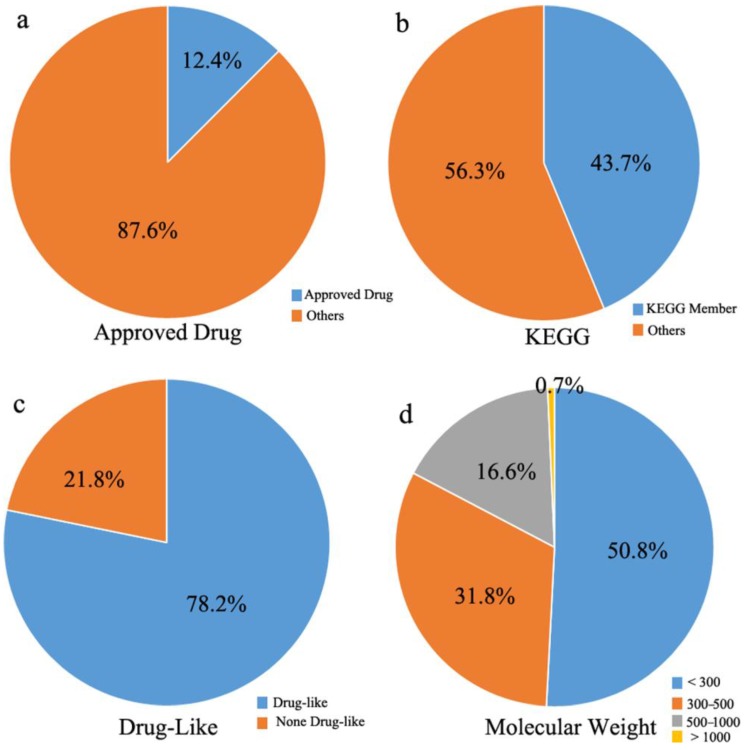
Statistical analyses for entries in the updated MTLD. (**a**) 304 (12.4%) entries are approved drugs from the DrugBank database; (**b**) 1069 (43.7%) entries belong to the Kyoto Encyclopedia of Genes and Genomes database; (**c**) 1911 (78.2%) entries are drug-like compounds according to Lipinski’s rule of five; (**d**) molecular weights of most ligands are ≤500 Da.

To better understand the constitution of the MTLs in the updated MTLD, a statistical analysis of the updated MTLD was performed, the results of which are shown in Figure 2. Firstly, in contrast to the known drugs listed in the DrugBank, 304 approved drugs were found in MTLD, about 12.4% of the overall entries (Figure 2a). Also, 1069 MTL entries in the MTLD also belonged to the KEGG database (a database of small molecules, biopolymers, and other chemical substances that are relevant to biological systems), which corresponds to about 43.7% of the overall entries (Figure 2b), and includes various amino acids, saccharides, nucleotides, and lipids. In particular, by using the module “QuaSAR-Descriptor”, included in Molecular Operating Environment (Chemical Computing Group, Montreal), according to Lipinski's rule of five, 1911 entries were predicted to be drug-like compounds (Figure 2c), corresponding to about 78.2% of the overall entries. The analysis of molecular weight distribution of MTLs in the MTLD indicated that most of them have molecular weights <500 Da, and a very small portion of the MTLs have a molecular weight >1000 Da (Figure 2d). Thus, the updated MTLD is quite similar to the previous version, based on statistical results. It suggests that updated MTLD could be highly relevant to the biological processes and drug action mechanism.

### 2.3. Multiple Conditional Search

**Figure 3:**
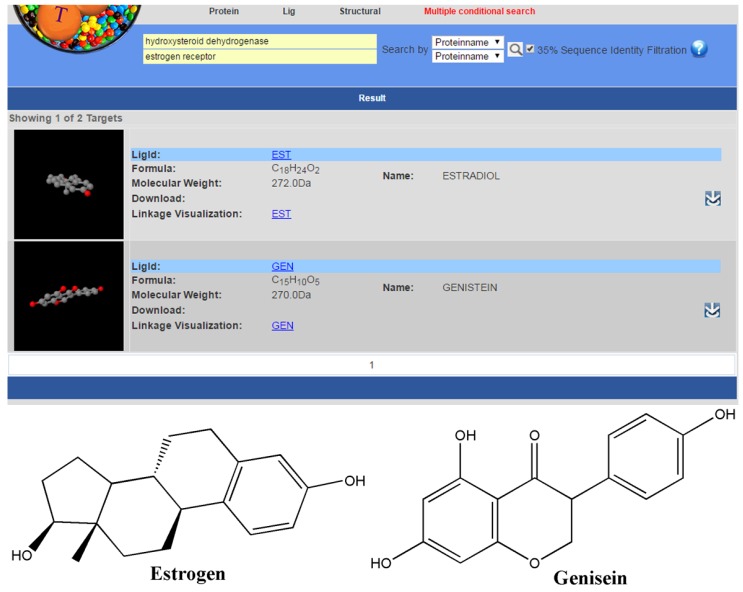
The multiple conditional search in MTLD.

To better identify the MTL that binds to the given multiple targets, we added another search mode “multiple conditional search”, in addition to the “Lig”, “Protein”, and “Structural” searches. Through “multiple conditional search”, we could conveniently find the MTL that binds to the target that we would like to query. For example, the estrogen receptor alpha is a current drug target for breast cancer. The 17-hydroxysteroid dehydrogenase (17HSD1) is a putative target for endocrine therapy of hormone-dependent breast cancer [17]. Through a multiple conditional search, we used the name of two proteins—estrogen receptor and hydroxysteroid dehydrogenase, respectively—as the key words for the multiple conditional search (Figure 3). As a result, estrogen (Lig-ID: EST) and genisein (Lig-ID: GEN) were obtained, which could provide an initial structure for the design of polypharmacological drugs that act on both targets. Otherwise, it was reported that flufenamic acid binds to androgen receptor and aldo-keto reductase family 1. Through querying using the multiple condition search (protein name: adrogen receptor and aldo-keto reductase family 1), flufenamic acid (lig-ID: FLF) was obtained, which was also found to bind with other targets, such as transthyretin, transcriptional enhancer factor TEF-4, and prostaglandin G/H synthase 2.

### 2.4. Linkage Visualization

**Figure 4:**
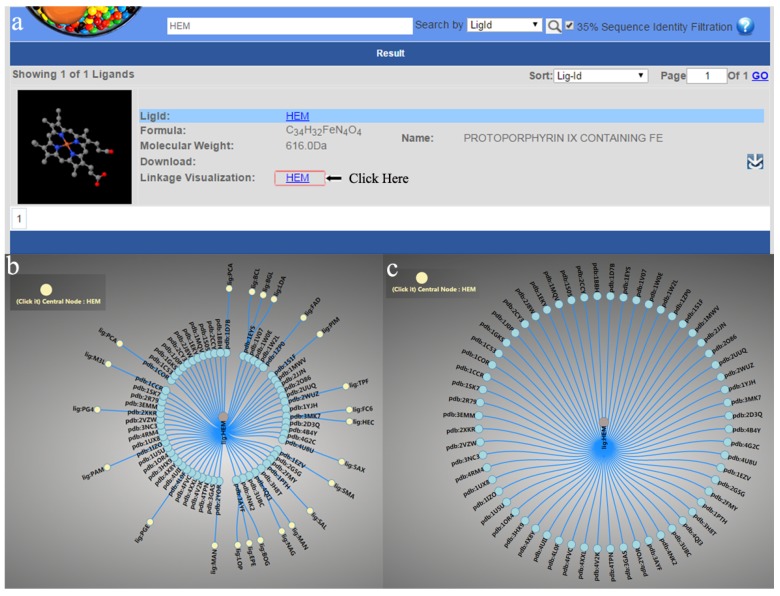
The Linkage visualization in MTLD. (**a**) The link for the linkage visualization; (**b**) two linkage layer mode visualization; (**c**) one linkage layer mode visualization.

When we analyzed the interaction between the MTL and its targets, we found that the interaction for some MTLs will somehow form an interaction network. Therefore, the “Linkage Visualization” function was added to depict such a network. For example (Figure 4), when we queried “HEM” in the “Lig” search using the “ligid” option, there was a “Linkage Visualization” link in the result page, and the interaction network could be visualized by clicking on the link (Figure 4a). The interaction network of HEM was shown in two linkage layers by default. Each solid circle in the network represented a ligand or a protein target, and the line between two circles indicates that there is an interaction between them (Figure 4b). When clicking the circle button at the left of the page, the network would switch into one linkage layer mode (Figure 4c). The brief information of the ligand or target can be shown by moving the mouse pointer over it. When clicking a circle in the network, a new network would be generated representing the selected circle as central. In particular, it is rather important to know that a compound interacts with two or more targets, as well as the strength of those interactions. As the bind affinity data between the ligands and the proteins are rather limited, we calculated the predicted affinities of the ligand and the protein based on the complex using x-score to further evaluate the specific binding or not. For each line in the linkage visualization map, the calculated x-score is shown beside the line to assess the binding affinity.

## 3. Discussion

Up to now, several data-sets of ligands that target multiple proteins have been mined from PDB for binding site similarity comparisons such as the Kahraman Dataset, Extended Kahraman Dataset, and Huang dataset [18,19]. The total MTL entries for these datasets together are no more than 100. Furthermore, recent ligand promiscuity analysis based on PDB generated two datasets, with 164 or 247 entries, respectively [14,15]. Recently, Zhang et al. also constructed a database (Polypharma, http:/imdlab.org/polypharma/) based on PDB, which contains 953 entries [20]. The current version of MTLD has 2444 MTLs. Compared with other existing databases of MTLs, it is the most comprehensive, detailed and complete. As a crucial expansion of the PDB, increasing numbers of MTLs will be included in the MTLD.

Altogether, the current version of MTLD includes 304 approved drugs and 1911 drug-like compounds, which may provide potential polypharmacological candidates. For instance, vemurafenib (Lig-ID: 032) is an effective B-RAF inhibitor that was developed for the treatment of late-stage melanoma [21]. By searching the MTLD, vemurafenib was found to bind to not only B-RAF [22], but also to the mixed lineage kinase ZAK [23], suggesting that vemurafenib may also provide therapeutic benefit through its off-target activity against ZAK [23]. Carprofen (Lig-ID: 0LA) is a non-steroidal anti-inflammatory drug, and it reduces inflammation by inhibition of COX-1 and COX-2 [24]. In the MTLD, carprofen was also seen to bind with the fatty acid amide hydrolase (FAAH) [25], which may guide the design of dual FAAH-COX inhibitors with superior analgesic efficacy [25]. Using the “Multiple conditional search”, it would be very convenient to obtain candidates that target the given multiple targets. Thus, the MTLD should be rather helpful in the development of polypharmacological drugs via provision of various possible candidates for further optimization. What is more, the binding modes of the candidate with given targets could be further generated according to their 3D structures to guide the optimization.

The linkage visualization function provides an alternative way to mine useful information from the MTLD apart from regular searching. Firstly, through the linkage visualization, it is possible to efficiently identify which targets a ligand may bind with, or with which ligands a target may be bound. What is more, it is possible to determine the relationship between the ligands in the linkage network. For instance, ligand1 and ligand2 bind with target1, while ligand2 also binds with target2, and they form a linkage network like “ligand1→target1←ligand2→target2”. As it is reported that the same ligand binds with different binding sites of different targets because of the similarity of the binding sites [14], it is very possible that ligand1 may also interact with target2. Therefore, more information will be mined via the linkage visualization function.

## 4. Materials and Methods

The original structural datasets for the updated MTLD were downloaded from PDB FTP archive (Version: June 2016) using the script “rsyncPDB.sh”. The data sets were automatically data-mined step by step using a program written in perl and c-shell.

Firstly, we sorted the protein structures from PDB (there are also DNA and RNA files included). Unlike the previous version, both X-ray and NMR protein structures from the PDB were considered for the extraction of ligands and their binding sites. Ligands containing >8 heavy atoms were extracted from selected PDB files. Binding sites were defined as all of the protein residues within a radius of 6.0 Å of each atom in binding ligands. Binding sites with >5 residues were outputted. To remove the redundancy of crystal structural entries bound to the same ligand, the sequence identity between protein pairs was restricted to <35%.

The web-server for the MTLD was constructed using the MySQL, Java, Javascript, and HTML languages on a machine with four 2.13 GHz processors. Several functions such as “Linkage Visualization” and “multiple conditional search” were also added for more convenient use of the database. The updated version of MTLD is a still free, internet-accessible database of MTLs, and the construction and interface is similar to the previous version.

## 5. Conclusions

Polypharmacology plays a rather important role in drug discovery. Herein, the MTLD was updated based on datasets extracted from the PDB. The updated MTLD comprises 2444 MTLs that bind to 21,424 binding sites from 18,231 PDB structures. In the updated MTLD, 304 entries are approved drugs, and 1911 entries are drug-like compounds. Thus, the MTLD could be extremely helpful for developing polypharmacological drugs. As a crucial expansion of the PDB results in increasing the numbers of MTLs, the MTLD will become an efficient platform for polypharmacological drug design.

## Figures and Tables

**Table 1:** A comparison of the updated MTLD with the previous version of MTLD.

Data-Sets	MTLD	MTLD_Updated	Increase Rate
MTL entries	1732	2444	41%
PDB structures	12,759	18,231	42.8%
Approved drugs	222	304	36.9%
Drug-like	1334	1911	43.2%
KEGG *	815	1069	31.1%

* KEGG: Kyoto Encyclopedia of Genes and Genomes database.
